# Ultrasound-based radiomics model for predicting molecular biomarkers in breast cancer

**DOI:** 10.3389/fonc.2023.1216446

**Published:** 2023-07-31

**Authors:** Rong Xu, Tao You, Chen Liu, Qing Lin, Quehui Guo, Guodong Zhong, Leilei Liu, Qiufang Ouyang

**Affiliations:** ^1^ Department of Ultrasound, The Second Affiliated Hospital of Fujian University of Traditional Chinese Medicine, Fuzhou, Fujian, China; ^2^ Department of Breast, The Second Affiliated Hospital of Fujian University of Traditional Chinese Medicine, Fuzhou, Fujian, China; ^3^ Department of Pathology, The Second Affiliated Hospital of Fujian University of Traditional Chinese Medicine, Fuzhou, Fujian, China

**Keywords:** radiomics, biomarker, breast cancer, ultrasonography, support vector machine

## Abstract

**Background:**

Breast cancer (BC) is the most common cancer in women and is highly heterogeneous. BC can be classified into four molecular subtypes based on the status of estrogen receptor (ER), progesterone receptor (PR), human epidermal growth factor receptor 2 (HER2) and proliferation marker protein Ki-67. However, they can only be obtained by biopsy or surgery, which is invasive. Radiomics can noninvasively predict molecular expression *via* extracting the image features. Nevertheless, there is a scarcity of data available regarding the prediction of molecular biomarker expression using ultrasound (US) images in BC.

**Objectives:**

To investigate the prediction performance of US radiomics for the assessment of molecular profiling in BC.

**Methods:**

A total of 342 patients with BC who underwent preoperative US examination between January 2013 and December 2021 were retrospectively included. They were confirmed by pathology and molecular subtype analysis of ER, PR, HER2 and Ki-67. The radiomics features were extracted and four molecular models were constructed through support vector machine (SVM). Pearson correlation coefficient heatmaps are employed to analyze the relationship between selected features and their predictive power on molecular expression. The receiver operating characteristic curve was used for the prediction performance of US radiomics in the assessment of molecular profiling.

**Results:**

359 lesions with 129 ER- and 230 ER+, 163 PR- and 196 PR+, 265 HER2- and 94 HER2+, 114 Ki-67- and 245 Ki-67+ expression were included. 1314 features were extracted from each ultrasound image. And there was a significant difference of some specific radiomics features between the molecule positive and negative groups. Multiple features demonstrated significant association with molecular biomarkers. The area under curves (AUCs) were 0.917, 0.835, 0.771, and 0.896 in the training set, while 0.868, 0.811, 0.722, and 0.706 in the validation set to predict ER, PR, HER2, and Ki-67 expression respectively.

**Conclusion:**

Ultrasound-based radiomics provides a promising method for predicting molecular biomarker expression of ER, PR, HER2, and Ki-67 in BC.

## Introduction

Breast cancer (BC) is currently the most prevalent form of cancer and is also the leading cause of cancer-related deaths among women, according to the International Agency for Research on Cancer (1). The four molecular biomarkers, namely estrogen receptor (ER), progesterone receptor (PR), human epidermal growth factor receptor 2 (HER2), and proliferation marker protein Ki-67, garner significant clinical attention in the clinical practice (2). These four molecular biomarkers play a crucial role in diagnosing BC. Based on the expression levels of these four molecular profiles (3), BC is classified into four distinct subtypes: luminal A, luminal B (including luminal B/HER2-negative and luminal B/HER2-positive), HER2-positive, and triple-negative BC (TNBC). In particular, the treatment protocols, prognosis, and metastatic potential of BC can vary significantly among these different molecular subtypes (4). Therefore, accurate prediction of the molecular profiles holds immense significance in guiding appropriate treatment strategies.

Currently, the assessment of molecular subtypes of BC before surgery typically relies on the results of immunohistochemistry (IHC) obtained through needle biopsy (5). However, this biopsy procedure is invasive and time-consuming. Additionally, a single local biopsy specimen may not always capture the complete molecular characteristics of the whole cancer, because of the high heterogeneity of BC (6). The tumor heterogeneity is an independent factor linked to the insufficient response to neoadjuvant chemotherapy (7). As a result, there is an urgent need for an alternative method that can accurately and non-invasively assess the expression of molecular biomarkers in BC.

With the rapid advancements in computer technology, the field of radiomics has emerged as a cutting-edge approach that harnesses high-throughput capabilities and mathematical algorithms to extract a wide range of quantitative features from medical images (8). This innovative technique not only overcomes the subjective limitations inherent in traditional imaging diagnosis but also enables a more comprehensive assessment of the overall characteristics of lesions and the surrounding tissue. Numerous studies have shown the effectiveness of radiomics based on X-ray, magnetic resonance imaging (MRI), ultrasound and positron emission tomography-computed tomography (PET-CT) for the evaluation of malignancy, differentiation of molecular subtype, and response to neoadjuvant therapy in BC (9). Ultrasound has unique advantages for clinical applications due to its real-time capabilities, frequent examination, and large data size. In particular, the US-radiomics model has demonstrated exceptional performance in distinguishing between benign and malignant breast lesions (10). However, despite these advantages, far few studies have investigated the application of ultrasound radiomics for predicting molecular biomarker expression (11). Furthermore, the number of studies exploring the specific radiomics features that hold great importance in predicting the molecular subtype of BC has been relatively limited.

In the present study, we investigated whether ultrasound radiomics features could be adopted as a predictive biomarker for discriminating the molecular biomarker profiling (ER, PR, HER2, and Ki-67). The purpose of this study was to explore the potential of radiomics features, and to provide complementary information to aid in the diagnostic molecular biomarker expression in BC.

## Methods

### Study design and cohort of the study

**Figure 1:**
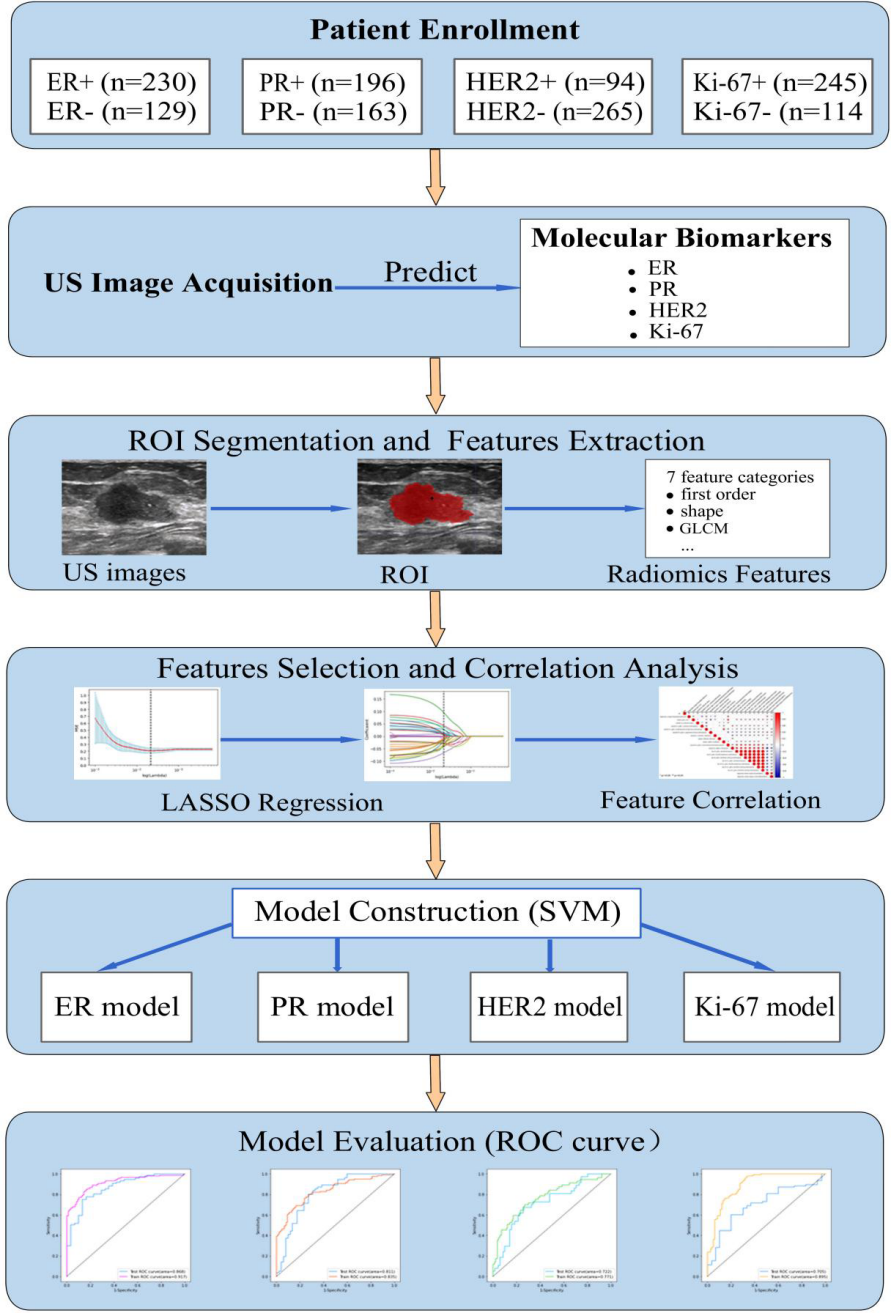
The workflow of this study. HER2, human epidermal growth factor receptor 2; TNBC, triple-negative breast cance; US, ultrasound; ER, estrogen receptor; PR, progesterone receptor; Ki-67, proliferating cell nuclear antigen; ROI, the region of interest; GLCM, gray level co-occurrence matrix; LASSO, least absolute shrinkage and selection operator; SVM, support vector machine; ROC, receiver operating characteristic.

This study was approved by the Ethics Committee of the Second Affiliated Hospital of Fujian University of Traditional Chinese Medicine (SPHFJP-T2022007-01), and informed consent was waived due to the retrospective nature of this study. We retrieved 466 consecutive patients with BC who underwent breast US examination and following treatment in our hospital from January 2013 to December 2021. Inclusion criteria were as follows: (1) Breast US was performed before the operation, and patients did not receive neoadjuvant chemotherapy (NAC) or biopsy prior to US examination; (2) Primary BC was confirmed by pathology; (3) Molecular subtype data (ER, PR, Ki-67, and HER2) were complete; (4) The US image quality met the diagnostic requirements. Exclusion criteria were as follows: (1) Patients without US examination; (2) Cases with incomplete pathological data; (3) Patients who had undergone local or systemic treatment such as puncture biopsy, chemotherapy, radiotherapy, ablation, or resection before breast US examination; (4) Cases with poor imaging quality. Finally, a total of 342 patients with invasive BC were included in this study. Among them, 341 were female and 1 was male. Their mean age was 54.5 years (range from 25 to 90 years old). The workflow of this work shown in **Figure 1** mainly includes six steps: patient enrollment, ultrasound image acquisition, features extraction, features selection, model construction and model evaluation.

### Breast ultrasonography

Breast ultrasound scanning was performed using Philips, GE, or Siemens color Doppler ultrasound equipment. The patients were positioned in a supine or lateral recumbent position with their hands raised to expose both breasts and axillae, allowing for a multi-angle scan to be performed. The lesions were scanned from multiple angles. And the largest section of ultrasound in each lesion was selected for analysis. The ultrasonic characteristics of the lesions were recorded, including their BI-RADS classification, location, size, shape, boundary, internal echo, calcification, posterior echo changes, blood flow, and axillary lymph nodes. The images were stored in DICOM format. The quality control of the images was carried out by two experienced radiologists, namely, Qing Lin and Quehui Guo. Both these experts possess proficiency in image analysis and worked in consensus to ensure the accuracy and reliability of this work.

### Pathology analysis

All primary breast lesions of the participants were pathologically confirmed by either biopsy or resection. Their expression levels of ER, PR, HER2, and Ki-67 were determined by IHC or fluorescence *in situ* hybridization. ER and PR positive is defined as more than 1%. For HER2, a score of 3+ indicated positive; + or no expression is negative; a score of 2+ requires FISH to determine the amplification status (12). The cutoff threshold for the Ki-67 is 20%. If Ki-67 is greater than or equal to 20%, it indicates highly proliferative and defines as positive (13). Based on the expression of ER, PR, HER2, and Ki-67, BC is divided into four molecular subtypes, i.e. luminal A, luminal B (including luminal B/HER2-negative and luminal B/HER2-positive), HER2-positive, and triple-negative.

### Segmentation of tumor and extraction of radiomics features

**Figure 2:**
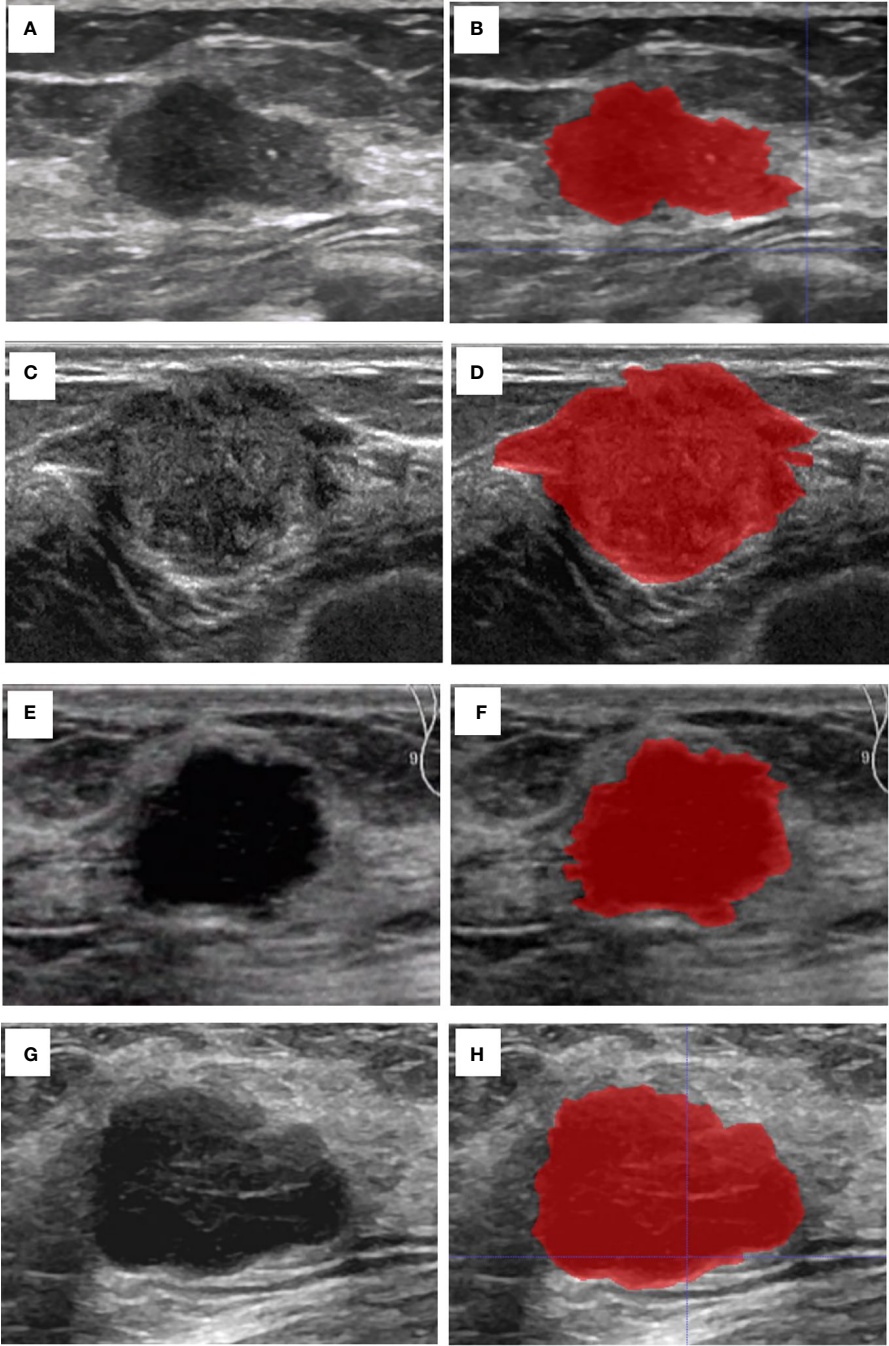
Cases of the original US image and the ROI. **(A)** The original US image of case 1 with invasive BC, Ki67 (5%+), ER (90%+), PR (60%+), and HER2 (-). **(B)** The ROI of case 1. **(C)** The original US image of case 2 with invasive BC, Ki-67 (30%+), ER (95%+), PR (95%+), and HER2 (-). **(D)** The ROI of case 2. **(E)** The original US image of case 3 shows a patient with invasive BC with myeloid characteristics, Ki67 (50%+), ER (-), PR (-), and HER2 (+). **(F)** The ROI of case 3. **(G)** The original US image of case 4 with invasive BC, Ki-67 (85%+), ER (-), PR (-), and HER2 (-). **(H)** The ROI of case 4.

The breast lesion region of interest (ROI) was manually designated on a grayscale ultrasound image by two sonographers. Those sonographers had no prior knowledge of the histopathological results. An open-source imaging platform, ITK-SNAP (http://www.itksnap.org), was utilized. To demonstrate the effectiveness of the ROI selection method, **Figure 2** displayed the original ultrasound image and the ROIs for four patients with breast carcinoma, each exhibiting different expression levels of molecular marker profile.

The extraction of lesion features was performed using Pyradiomics version 3.0 software. A total of 1314 radiomics features was extracted from each ultrasound image. Among these features, 7 categories of features were extracted: first order features (n = 252), shape features (n = 12), Gray Level Co-occurrence Matrix (GLCM, n = 336), Gray Level Run Length Matrix (GLRLM, n = 224), Gray Level Size Zone Matrix (GLSZM, n = 224), Gray Level Dependence Matrix (GLDM, n = 196), Neighboring Gray Tone Difference Matrix (NGTDM, n = 70).

### Features selection

The consistency of the extracted radiomics features was assessed with the inter- and intra-class correlation coefficient (ICC). Forty cases of ultrasound images, comprising 20 positive and 20 negative cases for each of the molecular biomarkers (ER, PR, HER2, and Ki-67), were randomly selected for analysis. To assess the reproducibility of the radiomics features, two experienced sonographers independently performed the ROI segmentation. Additionally, in order to evaluate inter-class reproducibility, sonographer 1 repeated the segmentation process one month after the initial ROI segmentation. Radiomics features with inter- and intra-class correlation coefficients (ICCs) greater than 0.75 were considered to demonstrate good reproducibility and were selected for model construction. Pearson’s coefficients matrix heatmaps were calculated to analyze the relationship between the radiomics features. And the most optimal features were selected.

### Construction of the radiomics model

Before proceeding with the modeling process, several data pre-processing steps were undertaken. These steps involved manual elimination of duplicate information, unpacking the multidimensional array into one-dimensional data by column, and filtering out features with zero variance using ANOVA. After standardizing the data, the least absolute shrinkage and selection operator (LASSO) logistic regression algorithm was used to select molecular-related features with non-zero coefficients, and the penalty parameters were tuned by 10-fold cross-validation. The mean and standard deviation of the selected features were calculated for both the negative and positive groups. The *t*-values and *P*-values were calculated to determine whether the features differed significantly between the two groups. The selected features were saved as radiomics labels for subsequent model construction.

The data were divided into a training set (70%) and a validation set (30%), with 251 and 108 lesions in the training and validation sets, respectively. Four support vector machine (SVM) models were created using the radiomics labels and the binary targets for ER, PR, HER2, and Ki-67. To optimize the performance of those models, the tree-structured Parzen Estimator (TPE), a hyperparameter optimization algorithm, was used.

### Evaluation of the model

To evaluate the diagnostic performance of the model on the training and validation sets, the receiver operating characteristic (ROC) curve was plotted, and the area under the curve (AUC) was calculated. Additionally, a confusion matrix was created to calculate the sensitivity, specificity, accuracy, and F1 score of the model.

### Statistical analysis

Python was used for statistical analysis (version 3.8.2). The normality and homogeneity of variance of the numeric data were assessed using the Kolmogorov-Smirnov test and F-test, respectively. The baseline characteristics for numeric variables was evaluated with the t-test, Fisher’s exact test, and MannWhitney U test. The Chi-square test was applied for categorical variables. A two-sided *p<* 0.05 was considered a significant difference. The statistical analysis packages include Levene, test, StandardScaler, MinMaxScaler, VarianceThreshold, train_test_split, cross_validate, cross_val_score, RepeatedKFold, confusion_matrix, accuracy_score, precision_score, recall_score, f1_score, roc_auc_score, roc_curve, LassoCV, SVC, and TPE. The Pearson’s coefficient was calculated using origin software.

## Results

### Clinicopathological characters

**Figure 3:**
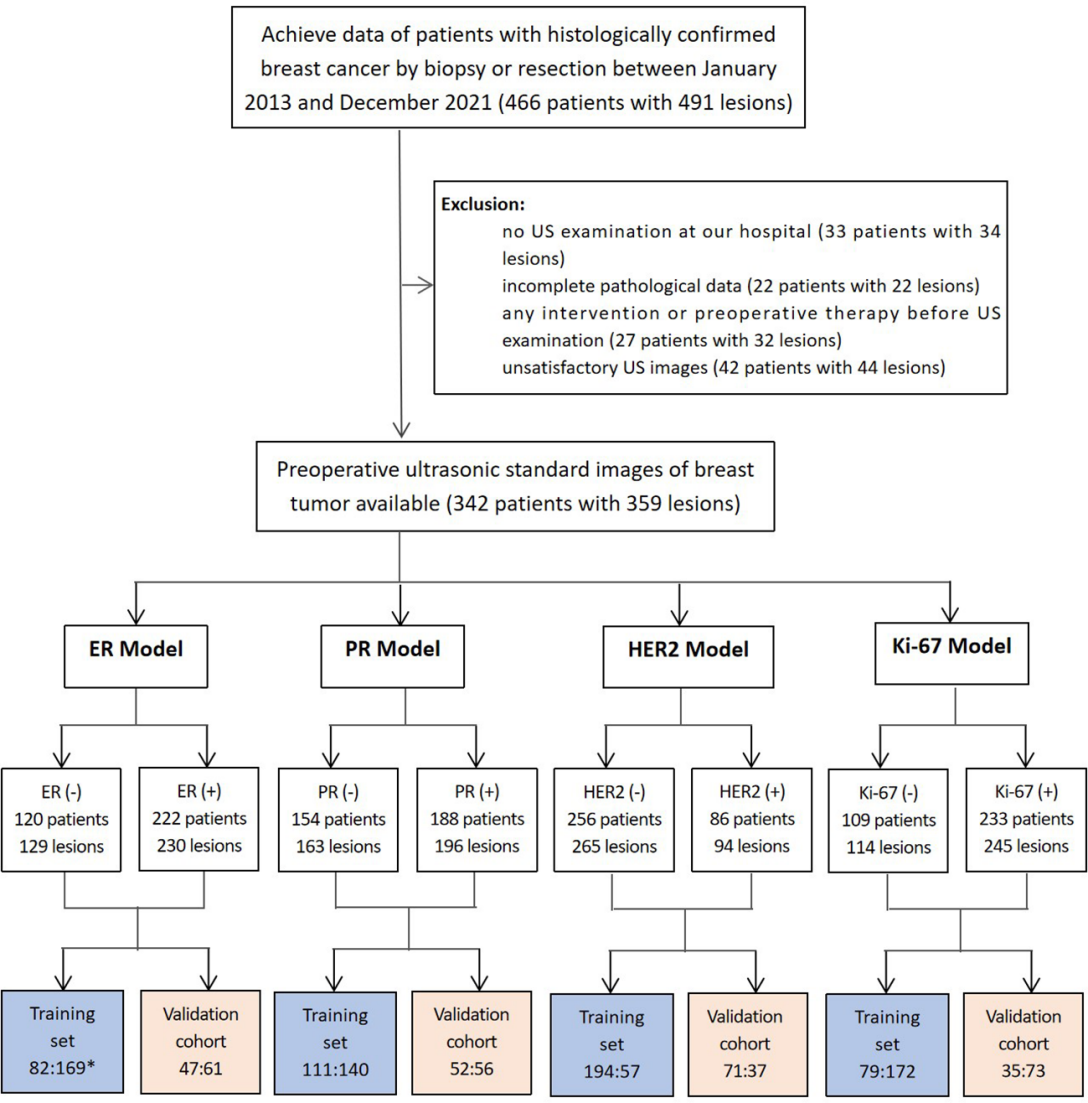
Patients included in this study (^*^comparison of the number of lesions in the negative and positive groups). 129 lesions were ER-negative and 230 were ER-positive. Similarly, 163 lesions were PR-negative while 196 were PR-positive. HER2 expression was negative in 265 lesions, while 94 lesions showed HER2-positive expression.

A total of 359 lesions were confirmed by pathology, with 326 cases (95.3%) having a single lesion, 15 cases (4.4%) two lesions, and 1 case (0.3%) three lesions. In terms of the histologic types, the most common type was invasive ductal carcinoma, accounting for approximately 70.5% (253 lesions), followed by the carcinoma in situ, accounting for 14.2% (51 lesions) and by the special types of invasive carcinoma, accounting for 13.1% (47 lesions). The clinicopathological characteristics of the patients were presented in **Table 1**; **Supplementary Table 2**. The distribution of molecular subtype was as follows: 86 were luminal A (24.0%), 146 were luminal B (40.7%), 63 were HER2+ (17.5%) and 64 were TNBC (17.8%). The baseline characteristics and clinicopathological information of both the training set and test set are summarized in **Table 2**. There were no significant differences in tumor size, age, gender, menopausal status, clinical staging, tumor types, molecular subtypes between the two groups. As demonstrated in **Figure 3**, the expression of ER, PR, HER2, and Ki-67 was as follows: 129 lesions were ER-negative and 230 were ER-positive. Similarly, 163 lesions were PR-negative while 196 were PR-positive. HER2 expression was negative in 265 lesions, while positive in 94 lesions. Moreover, Ki-67 expression was negative in 114 lesions, but positive in 245 lesions.

**Table 1:** Characteristics of the molecular biomarkers of patients.

Characteristics	All lesions	ER				PR					HER-2					Ki-67				
		(+)	(-)	*P*-value		(+)			(-)	*P*-value	(+)			(-)	*P*-value	(+)			(-)	*P-*value
age	54.5	53.9	54.9	0.484		53.7			55.0	0.305	53.0			54.7	0.228	53.8			55.4	0.231
Menopausal status				0.040						0.005`					0.783					0.826
Premenopausal	156	112	44			100			56		40			116		106			50	
Perimenopausal	7	5	2			5			2		1			6		4			3	
Postmenopausal	192	113	79			91			101		50			142		131			61	
Not available	4	0	4			0			4		3			1		4			0	
Tumor types				0.032						0.076					`0.005					0.015
Invasive ductal carcinoma	253	151	102			129			124		73			180		185			68	
Invasive lobular carcinoma	8	5	3			3			5		0			8		4			4	
The specific type of IC	47	38	9			30			17		4			43		29			18	
Carcinoma in situ	51	36	15			34			17		17			34		27			24	
^*^Histologic grade of IC				0.000						0.000					0.000					0.000
Grade I	23	22	1			19			4		1			22		8			15	
Grade II	122	97	25			82			40		20			102		71			51	
Grade III	133	59	74			47			86		49			84		120			13	
Grade X	30	16	14			14			16		7			23		19			11	
^#^Tumor classification of CIS				0.001						0.000					0.000					0.001
Group 1	8	8	0			8			0		0			8		1			7	
Group 2	19	17	2			17			2		2			17		7			12	
Group 3	24	11	13			9			15		15			9		19			5	

*Tumor grade of invasive cancer was divided into grade I (well differentiated), grade II (moderately differentiated), or grade III (poorly differentiated) according to the Scarff-Bloom-Richardson System. Grade X was defined as the grade that cannot be assessed or is unavailable. #Carcinoma in situ (CIS) cases were classified as group 1 (nonhigh grade CIS without comedo-type necrosis), group 2 (nonhigh grade CIS with comedo-type necrosis), or group 3 (high-grade CIS with or without comedo-type necrosis) according to the Van Nuys Classification. ER, estrogen receptor; PR, progesterone receptor; HER2, human epidermal growth factor receptor 2; Ki-67, proliferating cell nuclear antigen.IC, invasive carcinoma.

**Table 2:** Baseline characteristics comparison between the training set and test set.

Characteristics	Training set (n=251)	Test set (n=108)	*P*-value
Clinical tumor size (cm)			0.141
cT1 (≤ 2.0 cm)	128 (51.0%)	45 (41.7%)	
cT2 (2.1–5.0 cm)	109 (43.4%)	59 (54.6%)	
cT3 (> 5.0 cm)	14 (5.6%)	4 (3.7%)	
Age (years)	53.7	54.5	0.556
Gender			0.301
Female	251 (100%)	107 (99.1%)	
Male	0 (0%)	1 (0.09%)	
Menopausal status			0.397
Premenopausal	107 (42.6%)	49 (45.4%)	
Perimenopausal	138 (55.0%)	54 (50.0%)	
Postmenopausal	3 (1.2%)	4 (3.7%)	
Not available	3 (1.2%)	1 (0.9%)	
Clinical staging			0.092
Phase I	112 (44.6%)	35 (32.4%)	
Phase II	106 (42.2%)	58 (53.7%)	
Phase III	30 (12.0%)	15 (13.9%)	
Phase IV	3 (1.2%)	0 (0%)	
Tumor types			0.634
Invasive ductal carcinoma	172 (68.5%)	81 (75.0%)	
Invasive lobular carcinoma	6 (2.4%)	2 (1.9%)	
The specific type of IC	36 (14.3%)	11 (10.2%)	
Carcinoma in situ	37 (14.7%)	14 (13.0%)	
Molecular subtypes			0.391
Luminal A	58 (23.1%)	28 (25.9%)	
Luminal B	102 (40.6%)	44 (40.7%)	
HER2+	41 (16.3%)	22 (20.4%)	
Triple-negative	50 (19.9%)	14 (13.0%)	

TNM, Tumor node metastasis. IC, invasive carcinoma. DCIS, ductal carcinoma in situ. HER2, human epidermal growth factor receptor 2.

### Radiomics signature building

**Figure 4:**
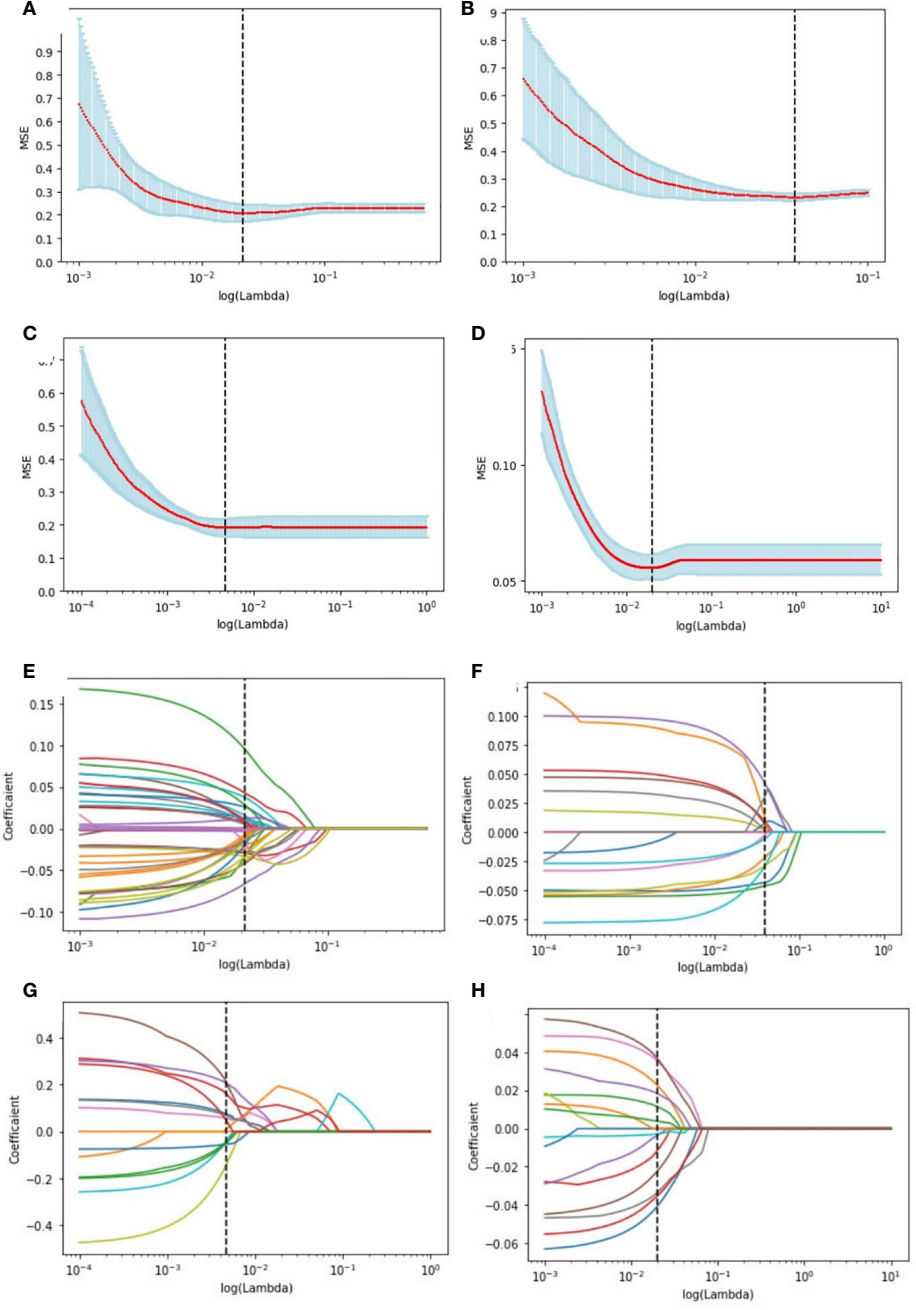
Radiomics feature selection using LASSO logistic regression in the primary cohort. Selection of the tuning parameter (λ) in the LASSO model of the ER **(A)**, PR **(B)**, HER2 **(C)**, and Ki-67 **(D)** *via* 10-fold cross-validation based on the mean standard error (MSE) of the minimum criteria. The value of Λ give the minimum average binominal deviance was used to select features. LASSO coefficient profiles of the selected radiomics features of the ER model **(E)**, PR model **(F)**, HER2 model **(G)**, and Ki-67 model **(H)**. Dotted vertical lines were drawn at the optimal values using the minimum criteria and the MSE criteria.

The study extracted 1314 features from each ultrasound image, and 1205 features were retained after processing. **Supplementary Table 1** shows the number of retained features after each step of feature selection. And the irrelevant features were removed. To select the relevant features, a LASSO logistic regression model was employed, then 39 and 20 signatures with non-zero coefficients were selected with the target of ER (**Figures 4A, E**) and PR (**Figures 4B, F**), respectively, in the primary cohort, after standardization. Normalization was applied before LASSO to choose the HER2-targeted signatures. And 14 signatures were selected by the LASSO algorithm (**Figures 4C, G**). Interestingly, no high-performance features were selected to classify Ki-67 binary data by 20% cutoff points, regardless of whether standardization or normalization was used before LASSO. Therefore, standardization was implemented, and LASSO was conducted on the Ki-67 target using continuous variables, specifically the exact values of the proliferation index, and 16 signatures were chosen (**Figures 4D, H**). The selected signatures were saved as radiomics labels for subsequent modeling.

### Correlation between the radiomics signature and molecular biomarkers

**Figure 5:**
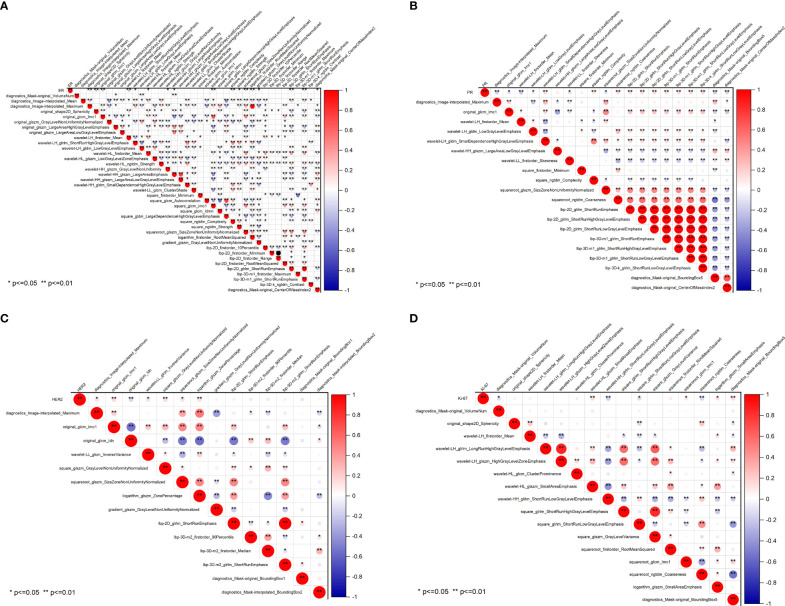
Pearson correlation coefficient heatmaps of selected features on predicting molecular expression of ER **(A)**, PR **(B)**, HER2 **(C)**, and Ki-67 **(D)**. Red represents positive correlations and blue indicates negative correlations. ER, estrogen receptor; PR, progesterone receptor; HER2, human epidermal growth factor 2; Ki-67, proliferating cell nuclear antigen.

The radiomics heatmap showcases a matrix of correlation coefficients among the features (**Figure 5**). The Pearson correlation coefficient was computed to evaluate the relationships among these features. The resulting heatmaps represents these associations, with the color red denoting positive correlations and the color blue indicating negative correlations.

To ensure the accuracy of the radiomics analysis, features with high correlation coefficients (r≥0.9) were removed from the initial pool of 1205 radiomics features. Only the features that exhibited a significant inter-group distribution difference were retained for further analysis. As a result, a total of 39 features were identified as essential for predicting ER expression, while 20 features for PR, 14 features for HER2, and 16 features for Ki-67. Notably, significant correlations are observed between the four molecular biomarkers and various radiomics features, including morphological features, grayscale features, texture features, and laws features.

### Radiomic features to predict molecular profiles

**Table 3** summarized the top five most significant features selected by the LASSO model, along with their corresponding *t*-values and *P*-values for the *t*-test. These values demonstrated a significant difference between the positive and negative groups (*P*<0.05). As compared to ER-negative cancer, ER-postive tumors had higher values of ShortRunEmphasis (SRE), Complexity, and ShortRunHighGrayLevelEmphasis (SRHGLE), while lower values of Imc1 and SizeZoneNonUniformityNormalized (SZNUN). Alternatively, PR-positive lesions showed higher values of SmallDependenceHighGrayLevelEmphasis (SDHGLE), while lower values of Maximum, SZNUN, BoundingBox5 and Imc1. HER2-postive cancers displayed significantly higher GrayLevelNonUniformityNormalized (GLNUN), SizeZoneNonUniformityNormalized (SZNUN), InverseVariance, ZonePercentage and Imc1, as compared with HER2-negative cancers. Ki-67-postive lesions showed higher BoundingBox5, SmallAreaEmphasis (SAE), while lower Coarseness, ShortRunLowGrayLevelEmphasis (SRLGLE) than Ki-67-negative cancers. Notably, SRE, Imc1, SZNUN, Complexity, Maximum, SDHGLE, BoundingBox5, GLNU, SRLGLE, and SAE were the most frequently selected signatures with significantly high weights (all *p<0.005*), indicating their importance in distinguishing between the positive and negative groups. They mainly belong to glcm, glrlm, glszm, ngtdm.

**Table 3:** The top five signatures were selected by Lasso and the *t*-test values.

Target	Top five features selected by Lasso	Features	Filter	$\bar{x}$ ± s		*t*-value	*P*-value
				Positive	Negative		
ER	ShortRunEmphasis	glrlm	lbp-2D	0.109 ± 0.069	0.086 ± 0.052	3.341	0.0009
	Imc1	glcm	original	-0.198 ± 0.089	-0.164 ± 0.075	3.660	0.0003
	SizeZoneNonUniformityNormalized	glszm	squareroot	0.466 ± 0.107	0.502 ± 0.113	3.003	0.0029
	Complexity	ngtdm	square	3.261 ± 5.484	1.735 ± 3.168	2.900	0.0040
	ShortRunHighGrayLevelEmphasis	glrlm	wavelet-LH	59.517 ± 60.040	44.905 ± 32.122	2.565	0.0107
PR	Maximum	Image-interpolated	diagnostics	387.129 ± 124.209	438.003 ± 142.456	3.614	0.0003
	SmallDependenceHighGrayLevelEmphasis	gldm	wavelet-LH	22.668 ± 26.959	15.418 ± 12.397	3.165	0.0017
	SizeZoneNonUniformityNormalized	glszm	squareroot	0.461 ± 0.125	0.494 ± 0.112	2.485	0.0134
	BoundingBox5	Mask-original	diagnostics	172.755 ± 87.278	204.883 ± 82.722	3.556	0.0004
	Imc1	glcm	original	-0.196 ± 0.089	-0.173 ± 0.079	2.561	0.0109
HER2	GrayLevelNonUniformityNormalized	glszm	square	0.493 ± 0.206	0.429 ± 0.179	2.878	0.0042
	SizeZoneNonUniformityNormalized	glszm	squareroot	0.503 ± 0.109	0.470 ± 0.109	2.524	0.0120
	InverseVariance	glcm	wavelet-LL	0.418 ± 0.052	0.399 ± 0.065	2.467	0.0140
	ZonePercentage	glszm	logarithm	0.484 ± 0.167	0.429 ± 0.176	2.654	0.0083
	Imc1	glcm	original	-0.163 ± 0.075	-0.193 ± 0.088	2.996	0.0029
Ki-67	Coarseness	ngtdm	squareroot	0.021 ± 0.022	0.029 ± 0.032	2.802	0.0054
	BoundingBox5	Mask-original	diagnostics	195.792 ± 86.362	169.184 ± 84.711	2.734	0.0066
	ShortRunLowGrayLevelEmphasis	glrlm	wavelet-HH	0.088 ± 0.062	0.115 ± 0.091	3.213	0.0014
	SmallAreaEmphasis	glszm	wavelet-HL	0.604 ± 0.078	0.575 ± 0.106	2.911	0.0038
	ShortRunLowGrayLevelEmphasis	glrlm	square	0.154 ± 0.061	0.172 ± 0.067	2.490	0.0132

ER, estrogen receptor; PR, progesterone receptor; HER2, human epidermal growth factor receptor 2; Ki-67, proliferating cell nuclear antigen.

### SVM model construction and validation of the model

**Figure 6:**
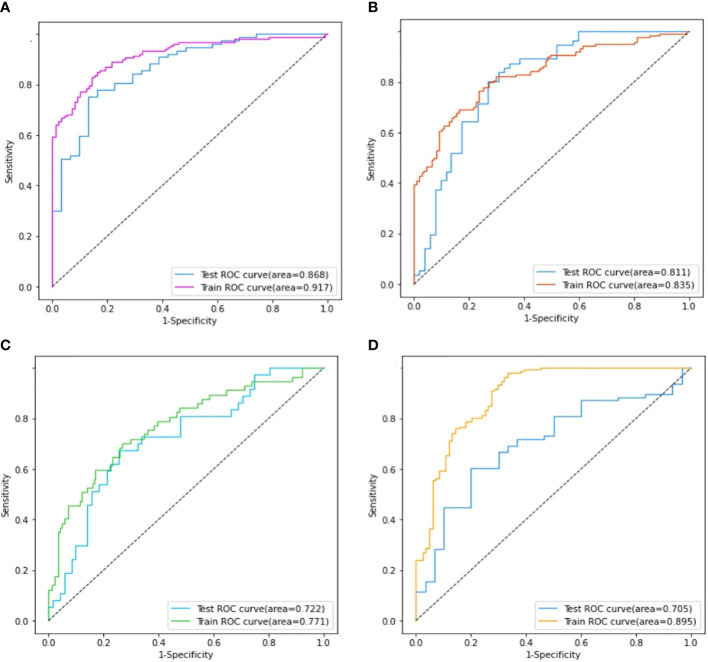
Comparison of the area under the ROC curves on the training set and validation cohort of ER model **(A)**, PR model **(B)**, HER2 model **(C)**, and Ki-67 model **(D)**.

Four models for predicting the molecular biomarkers of ER, PR, HER2, and Ki-67 were created using the features selected by LASSO and the parameters optimized by TPE. Subsequently, four ROC curves were plotted to evaluate the diagnostic efficacy of the models. The AUCs for the training and validation cohorts were presented in **Figure 6**. The diagnostic efficacy of the four ROC curves was ranked that ER model being the most effective, followed by the PR model, HER2 model, and lastly the Ki-67 model.

The performance of the four models namely the ER model, PR model, HER2 model, and Ki-67 model, were evaluated. Those assessment parameters, including sensitivity, specificity, accuracy, and F1 score are presented in **Table 4**. The ultrasound-based radiomics model displayed the highest discriminatory power for ER, achieving an AUC of 0.917 in the training set and 0.868 in the validation cohort (**Figure 6A**). For PR, the radiomics model achieved an AUC of 0.835 in the training set and 0.811 in the validation cohort (**Figure 6B**). The radiomics model generated an AUC of 0.722 (**Figure 6C**) and 0.706 (**Figure 6D**) for HER2 and Ki-67 in the validation cohort, respectively, which was slightly lower than those for ER and PR. Those results suggest that all four models are effective in predicting the molecular expression of BC. Notably, the degree of model fitting for ER, PR, and HER2 exhibited remarkable performance, with no significant signs of overfitting. Conversely, overfitting was evident for Ki-67.

**Table 4:** Diagnostic Performances of the SVM model.

target	cohort	AUC	%Sn	%Sp	%Acc	F1 score
ER	train	0.917	77.1	89.8	82.1	0.840
	validate	0.868	75.3	87.1	78.7	0.835
PR	train	0.835	69.3	81.1	74.5	0.752
	validate	0.810	78.6	73.1	75.9	0.772
HER2	train	0.771	71.9	70.1	70.5	0.526
	validate	0.722	73.0	66.2	68.5	0.614
Ki-67	train	0.896	73.3	83.5	76.5	0.810
	validate	0.706	61.6	77.1	66.7	0.714

support vector machine, SVM; area under the curve, AUC; sensitivity, Sn; specificity, Sp; accuracy, ACC; ER, estrogen receptor; PR, progesterone receptor; HER2, human epidermal growth factor receptor 2; Ki-67, proliferating cell nuclear antigen.

## Discussion

Molecular subtyping plays a vital role in tailoring treatment approaches to individual patients. However, it requires biopsy or surgery which is invasive, time-consuming, and sometimes prone to inaccurate due to the heterogeneity. In recent studies, radiomics shows good performance for predicting molecular subtypes of BC (14). In our study, we extracted ultrasound radiomics features to build the prediction models for the expression of ER, PR, HER2, and Ki-67 in BC. Our results indicate that the ultrasound-based radiomics models show excellent performance in predicting molecular biomarkers in BC. Additionally, our research identified several critical radiomics features that play a substantial role in distinguishing between positive and negative expressions of molecular biomarkers. These features, namely SRE, Imc1, SZNUN, Complexity, Maximum, SDHGLE, BoundingBox5, GLNU, SRLGLE, and SAE are highly associated with the expression of ER, PR, HER2, and Ki-67. It is noteworthy that, to the best of our knowledge, our study is the first to establish a relationship between ultrasound-based radiomics features and molecular profiles. Our study offers a non-invasive, cost-effective, and time-efficient alternative for BR molecular classification. And the identification of these specific features provides valuable insights for further research and potential development of diagnostic tools.

It is well-known that the aggressiveness of BC is closely related to its heterogeneity (15, 16), which sometimes is challenging to assess fully when using histopathological tissue samples obtained from needle biopsies (17, 18). The accuracy of molecule profiling diagnosis can be impacted by the size and number of samples obtained (19). Radiomics is a powerful tool that enables the non-invasive assessment of whole-tumor heterogeneity by extracting quantitative features based on texture, shape, and intensity (20). These features provide valuable insights into the underlying biological processes of the imaged tissue, including tumor heterogeneity, microenvironmental characteristics, and etc. There is a growing literature that has reported to predict molecular profiling in BC, but mostly based on MRI and X-ray analysis (21, 22). However, there has been a limited number of studies conducted thus far that utilize ultrasound imaging as the primary modality for investigation (23).

Radiomics features are quantitative descriptors that encompass various aspects of a medical image, including intensity, shape, volume, texture, and etc. They are usually difficult to be interpreted and analyzed intuitively. In our study, 7 categories of image features were extracted from the 1314 radiomics features. We have innovatively developed four molecular prediction models based on ultrastructural features. In the ER-positive model, higher values were observed for SRE, Complexity, and SRHGLE, while lower values were found for Imc1 and SZNUN. Similarly, in the PR-positive model, higher values were observed for SDHGLE, while lower values were found for Maximum, SZNUN, BoundingBox5, and Imc1. The HER2-positive model displayed significantly higher values for GLNUN, SZNUN, InverseVariance, ZonePercentage, and Imc1 compared to HER2-negative BC. In the Ki-67-positive model, higher values were observed for BoundingBox5 and SAE, while lower values were found for Coarseness and SRLGLE, compared to Ki-67-negative lesions. The features of SRE, Imc1, SZNUN, Complexity, Maximum, SDHGLE, BoundingBox5, GLNU, SRLGLE, and SAE are heavily weighted (all *P<0.005*), indicating their pivotal role in discerning the negative or positive expression of ER, PR, HER2, and Ki-67 molecules. SRE can assess the distribution of short runs of similar intensity values within an image, which can characterize the texture of BC. Its higher values mean a greater proportion of short runs of similar intensity values in the image. Imc1 can characterizes the similarity of gray-level intensity values between adjacent pixels, taking into account their relative positions. SZNUN can determine the degree of heterogeneity in the sizes of homogeneous regions within an image. Its higher values indicate greater variability in the sizes of homogeneous regions across the image. Complexity characterizes the heterogeneity and irregularity in the image intensity values. And the higher values indicating greater complexity and heterogeneity in the image. Maximum is to measure the maximum intensity value in the interpolated image. Complexity characterizes the heterogeneity and irregularity in the image intensity values. And the higher values indicating greater complexity and heterogeneity in the image. Maximum is to measure the maximum intensity value in the interpolated image. SDHGLE measures the joint probability of occurrence of small dependence gray level values with high gray-level values. It can characterize the heterogeneity of a tumor. BoundingBox5 characterizes the compactness of ROI in an image, with higher values indicating that the ROI is more compact. GLNU quantifies the degree of variation in gray-level intensity. A higher value of GLNU indicates that the intensity values within the ROI are more widely distributed, suggesting higher degree of heterogeneity. SRLGLE quantifies the small runs of low gray-level values within an image. SAE measures the proportion of small homogeneous areas in the image, with a higher value indicating a greater proportion of small, homogeneous areas. To the best of our knowledge, this is the first study to investigate the correlation between the aforementioned radiomics features and molecular biomarkers. Their heavy weight emphasizes their importance as crucial markers in the assessment of molecular expression.

These features, including GLCM, GLRLM, GLSZM, and NGTDM, mainly belong to the categories of second-order statistics or higher-order statistics. They provide valuable insights into the irregular or heterogeneous texture of tumors that are not discernible to the naked eye. As far as we know, there are very few studies on the correlation between the aforementioned radiomics features and molecular biomarkers. Previous report indicated that higher Ki-67 expression was associated with posterior acoustic enhancement, and P53-positive cancer was associated with an absence of anecho halo, which was different from ours (24). This inconsistency may be due to the different feature extraction methods. The presence of irregular or heterogeneous tumor textures, as indicated by these features, holds significant clinical implications. It suggests the presence of diverse tissue components within the tumor, potentially reflecting variations in cellularity, vascularity, and spatial organization.

The SVM models created based on the LASSO-selected features and PET-optimized parameters can identify molecular indicators effectively. Our results indicate that US-based radiomics models show optimal performance to predict molecular profiling, with the best for ER, and followed by PR. Both of them had an AUC greater than 0.80 in the validation cohort, whereas they showed lower diagnostic efficacy for HER2 and Ki-67, with an AUC slightly higher than 0.70 in the validation cohort. The ER model performed well in the validated cohort with a high specificity of 87.1% and an F1 score of 0.835. Before modeling, the choice of normalization and the setting of LASSO parameters is crucial, as both will affect the quantity and quality of LASSO feature selection. Moreover, the effectiveness of features will greatly influence the model’s validity. The AUCs for predicting molecular subtype we achieved are similar to the AUCs of 0.74–0.97 in the other literature (25, 26).

In recent years, deep learning techniques have been widely employed to investigate the molecular expression of BC (27, 28). Deep learning models have demonstrated superior diagnostic performance compared to traditional machine learning models. However, deep learning models require a relatively larger sample size than traditional machine learning approaches. Additionally, the training process of deep learning models can be likened to a “blind box,” making it challenging to discern which features are utilized in the modeling process and how they are interconnected. In contrast, machine learning models offer interpretability by enabling the analysis of specific features and their corresponding weights throughout the modeling process (29, 30).

Our study has certain limitations that should be acknowledged. Firstly, it is based on a retrospective, single-center design, and the sample size is relatively small. Therefore, caution should be exercised in generalizing the findings to larger populations. To validate and strengthen our results, further investigations using a larger, multi-center cohort are warranted. Another limitation of our study is the utilization of only two-dimensional grayscale data. The inclusion of additional imaging modalities or three-dimensional data could provide a more comprehensive assessment of the molecular profiling in BC. Additionally, research in the series including the prediction of molecular subtypes, clinical decision making or therapy response based on radiomics would enhance the reliability and value of the radiomics analysis. Despite these limitations, our findings hold significant value and contribute to the understanding of the potential of ultrasound radiomics in assessing the molecular characteristics of BC.

## Conclusions

Our study provides evidence that some specific radiomics features extracted from ultrasound images can effectively predict molecular expression of ER, PR, HER2, and Ki-67 in BC. The radiomics models based on the selected radiomics features show good performance in non-invasively assessing the molecular subtypes. Our findings provide a promising method in assessing the molecular profile of breast cancer.

## Data availability statement

The original contributions presented in the study are included in the article/**Supplementary Material**, Further inquiries can be directed to the corresponding authors.

## Ethics statement

The studies involving human participants were reviewed and approved by the Ethics Committee of the Second Affiliated Hospital of Fujian University of Traditional Chinese Medicine (SPHFJP-T2022007-01). The ethics committee waived the requirement of written informed consent for participation. Ethical review and approval was not required for the animal study because There were no animal experiments in this study and no animal ethics is required. Written informed consent was obtained from the individual(s) for the publication of any potentially identifiable images or data included in this article.

## Author contributions

RX contributed to concept development, literature searching, and writing original draft. TY contributed to the software using, and language editing. CL contributed to the patient enrollment. QL and QG contributed to the imaging processing, and methodology. GZ contributed to the analysis of pathology. LL contributed to study management, statistical analysis and methodology. QO contributed to the funding acquisition, and study management. All authors contributed to the article and approved the submitted version.
